# Clinical-Oriented Hierarchical Machine Learning Framework for Early Kidney Tumor Detection and Malignant Subtype Classification

**DOI:** 10.3390/tomography11110122

**Published:** 2025-10-30

**Authors:** Mansourah Aljohani

**Affiliations:** Department of Computer Science, College of Computer Science and Engineering, Taibah University, Yanbu 40292, Saudi Arabia; mjohni@taibahu.edu.sa

**Keywords:** computed tomography (CT), deep learning (DL), embeddings, kidney tumor detection, radiomics

## Abstract

**Objectives:** Kidneytumors, particularly renal cell carcinoma (RCC), represent a critical public health concern due to their prevalence and the severe consequences of late diagnosis. Traditional diagnostic techniques, though widely used, are often limited by human error, inter-observer variability, and delayed recognition of malignant subtypes, underscoring the urgent need for automated, accurate, and reproducible solutions. **Methods:** To address these challenges, this study introduces a hierarchical, AI-driven framework for early detection and precise classification of kidney tumors from CT scans. At its core, the framework uses a specialized encoder, RAD-DINO-MAIRA-2, to extract highly discriminative imaging features, which are subsequently processed through multiple machine learning classifiers tailored to distinct hierarchical levels of diagnosis. **Results:** Using benchmark kidney tumor datasets, the framework was rigorously validated across 25 independent trials. Performance was assessed using accuracy, reproducibility, and robustness metrics, with results revealing a maximum accuracy of 98.29% and a mean accuracy of 94.72%. Notably, the Gaussian Process classifier achieved perfect performance in tumor type classification, while the MLP classifier attained flawless results in malignant subtype differentiation. Comparative analyses demonstrate that our hierarchical approach outperforms conventional DL-based pipelines by reducing sensitivity to dataset variability and providing a clinically viable path for integration into diagnostic workflows. Combining state-of-the-art feature extraction with hierarchical classification, the proposed framework delivers a robust and interpretable tool with substantial promise for improving patient outcomes in real-world clinical practice.

## Simple Summary

Kidney cancer is easier to treat when it is diagnosed early, but reading CT scans and spotting small tumors can be challenging. This study introduces an intelligent computer system that helps doctors detect kidney tumors and identify whether they are harmless or cancerous. The system was trained on real CT scans from hospital patients and showed excellent accuracy in finding and classifying tumors. This approach could support radiologists in making quicker, more confident decisions and encourage future research on using artificial intelligence in everyday medical care.

## 1. Introduction

The kidneys (two bean-shaped organs situated in the lower abdominal region) play a vital role in preserving the body’s internal balance. They remove waste materials, surplus fluids, and harmful substances from the bloodstream, control blood pressure, maintain electrolyte equilibrium, and generate hormones that aid in red blood cell formation and bone health [1]. When kidney function is compromised, it can lead to severe systemic consequences, including chronic kidney disease, hypertension, and even organ failure [2,3].

Kidney tumors, which arise from the abnormal growth of cells within the kidney, are a significant threat to kidney health. These tumors can impair kidney function, cause pain, and, if malignant, metastasize to other organs, leading to life-threatening complications [4,5]. The most common type of kidney tumor is renal cell carcinoma (RCC), which accounts for ≈90% of all kidney cancers [6]. Other subtypes include clear cell, papillary, and chromophobe RCC and benign lesions such as angiomyolipomas and oncocytomas [7,8].

According to Lee et al., kidney cancer ranks as one of the top 10 most commonly diagnosed cancers in the US, with an estimated 76K new cases and 13,780 fatalities recorded in 2021 [9,10]. Over the past five years, the incidence of kidney tumors has steadily increased, with a 1.1% annual rise in new cases, highlighting the growing burden of this disease [11]. Timely identification and precise differentiation of kidney tumors are essential for successful treatment and better patient prognoses. However, the heterogeneity of kidney tumors, integrated with their often asymptomatic nature in early stages, poses significant diagnostic challenges [12,13].

Several imaging modalities are commonly used to diagnose kidney tumors, each with its strengths and limitations [14,15]. Computed tomography (CT) is the primary imaging modality due to its high spatial resolution and ability to provide detailed anatomical information [16]. It is particularly effective for detecting small tumors and assessing tumor staging, as revealed by Sasaguri and Takahashi (2018), who highlighted its role in characterizing solid renal masses [17]. Magnetic resonance imaging (MRI) is often used as a complementary modality, especially for complex cases or when CT findings are inconclusive, as it provides exceptional soft tissue contrast and avoids the use of ionizing radiation [18].

Ultrasound is widely used as an initial screening tool due to its non-invasive nature, cost-effectiveness, and ability to differentiate solid masses from cysts [18]. Contrast-enhanced ultrasound (CEUS) and CT urography (CTU) are also employed for more detailed assessments, particularly in assessing tumor vascularity and urinary tract abnormalities [19,20]. In advanced cases, positron emission tomography (PET-CT) is used to evaluate tumor metabolism and detect metastases. At the same time, multiparametric MRI (mpMRI) provides a comprehensive assessment of tumor heterogeneity and predicts histology, as supported by Yap et al. and LoCastro et al. [21,22].

Although these imaging techniques are accessible, conventional diagnostic methods depend significantly on radiologists’ manual assessments, which are labor-intensive, susceptible to errors, and frequently inconsistent due to the subjective interpretation of images [23,24,25]. One of the significant challenges in conventional approaches is intra-observer variability, where the same radiologist may interpret the same image differently at different times, leading to inconsistencies in diagnosis [26,27]. Additionally, inter-observer variability, where radiologists may have differing interpretations of the same image, further complicates the diagnostic process [26,27]. These limitations are exacerbated by the increasing volume of medical imaging data, leading to delays in diagnosis and potential misclassification of tumors. As a result, there is a pressing need for automated systems that can enhance kidney tumor diagnosis efficiency, accuracy, and consistency [28].

Artificial intelligence (AI) (selectively deep learning (DL)) has become apparent as a transformative tool in medical imaging, offering the potential to automate and improve the diagnostic process [29]. AI-based systems can analyze large datasets of medical images, extract complex patterns, and provide quantitative assessments beyond human capability [30]. AI has shown promise in tasks such as tumor detection, classification, and segmentation [31]. For example, Zhou et al. revealed the potency of convolutional neural networks (CNNs) in differentiating between benign and malignant renal masses on CT scans, achieving high accuracy rates [32]. These advancements highlight the potential of AI in addressing the limitations of conventional diagnostic approaches, including intra-observer and inter-observer variability [33].

Research Gaps: Despite notable progress in applying deep learning (DL) and hybrid machine learning (ML) models for kidney tumor detection and classification, existing approaches still face several persistent challenges that hinder their translation into routine clinical practice. Prior studies (e.g., Praveen et al. [34], Alzu et al. [35], and Almuayqil et al. [36]) have reported improvements in accuracy and computational efficiency; however, issues related to data diversity, robustness, and clinical usability remain unresolved. These limitations highlight the need for a more reliable, reproducible, and hierarchically structured diagnostic framework. The key gaps identified are as follows:

- Dataset variability and bias: Many models are evaluated on limited or homogeneous datasets, reducing generalizability across diverse patient populations.
- Model robustness and reproducibility: Reported accuracies fluctuate across trials, with insufficient emphasis on reproducibility and stability of results.
- Clinical integration barriers: Most existing frameworks are not designed for real-time or workflow-compatible applications, limiting clinical adoption.
- Granularity of classification: Current studies often overlook malignant subtype differentiation, essential for treatment planning and prognosis.
- Interpretability and trust: Few approaches incorporate interpretable designs that can provide clinicians with transparent, trustworthy decision support.

Objective: Therefore, the primary objective of this study is to develop a robust, reproducible, and clinically viable hierarchical AI framework for early kidney tumor detection and malignant subtype classification, specifically designed to overcome the limitations of dataset variability, model robustness, clinical integration barriers, granularity of classification, and interpretability identified in current approaches.

Contributions: To achieve this objective, this study makes the following key contributions:

- Developing a hierarchical AI-driven kidney tumor detection and classification framework.
- Utilization of a specialized encoder (RAD-DINO-MAIRA-2) for feature extraction from CT scans.
- Evaluation of multiple ML classifiers to identify the best-performing model for each hierarchical level.
- Demonstration of the framework’s potential for integration into clinical workflows, improving diagnostic accuracy and patient outcomes.

The manuscript’s structure is as follows: Section 2 reviews relevant studies. Section 3 presents the materials/dataset. Section 4 explains the methodology applied. Section 5 covers the experimental design, findings, and an in-depth analysis of the results. Lastly, Section 6 provides concluding remarks and explores avenues for future research.

## 2. Related Studies

Recent advancements in DL have significantly improved the detection and classification of kidney tumors, as revealed by several studies. Praveen et al. [34] addressed the global health risk of renal neoplasms by developing a robust detection model using a fusion of ResNet and ResNeXt architectures. Their approach used the multi-path network capabilities of ResNeXt to enhance gradient flow and scalability, achieving an impressive 94% accuracy on a subset of 2170 images from the KAUH dataset. This study highlights the potential of combining advanced CNN architectures to improve diagnostic accuracy and healthcare outcomes.

Building on this, Alzu et al. [35] proposed a comprehensive kidney tumor detection and classification framework using 2D-CNN models, including CNN-6, ResNet50, and VGG16. Their work utilized a novel dataset of 8400 CT scan images from 120 patients, achieving accuracies of 97%, 96%, and 60% for the detection models and 92% for the classification model. These results underscore the potency of DL in reducing radiologists’ workload and improving diagnostic precision, particularly in early detection, which is critical for patient outcomes.

Similarly, Qadir et al. [37] explored the application of AI in diagnosing kidney diseases, including tumors, cysts, and stones. Their study employed a hybrid approach, combining the Densenet-201 model for feature extraction with Random Forest for classification. This method achieved a remarkable accuracy of 99.719% on a dataset of 12,446 CT urogram and whole abdomen images, demonstrating the potential of integrating pre-trained models with ML algorithms for highly accurate kidney disease diagnosis.

Kaur et al. [38] further advanced the field by developing a Sequential CNN model for tumor detection in medical images. Their study used a preprocessed and augmented dataset of 8400 images to ensure balanced classes. The model achieved a training accuracy of 97.69% and a validation accuracy of 95.31%, with high precision and recall values of 0.97 for both classes. This research highlights the potency of CNNs in automated diagnostic systems and suggests that future work could explore more advanced architectures and larger datasets to enhance performance further.

Finally, Almuayqil et al. [36] introduced KidneyNet, a state-of-the-art CAD system for diagnosing chronic kidney diseases, including tumors, cysts, and stones. KidneyNet’s CNN architecture, integrated with the Grad-CAM algorithm for interpretability, achieved exceptional performance metrics, including 99.88% accuracy, 99.92% specificity, and 99.76% sensitivity. This system improves diagnostic accuracy and provides clinicians with actionable insights by highlighting affected areas in CT scans, promoting early detection, and reducing the diagnostic burden on radiologists.

A critical examination of the existing body of research on kidney tumor detection and classification reveals that, despite notable advances in deep learning and hybrid machine learning methodologies, several persistent shortcomings impede their clinical adoption. Most prior studies emphasize algorithmic accuracy while overlooking robustness, reproducibility, interpretability, and clinical workflow integration. Furthermore, limited dataset diversity and the absence of structured, hierarchical diagnostic pipelines have restricted the generalizability of results across real-world patient populations. The present study introduces a clinically aligned, hierarchical AI framework to bridge these gaps to ensure diagnostic transparency, multi-level classification, and integration feasibility within clinical environments. Table 1 concisely maps the identified research gaps and the corresponding contributions proposed in this work.

## 3. Materials

The current study utilized a dataset of renal CT scans from the King Abdullah University Hospital (KAUH) in Jordan, comprising 8400 images from 120 adult patients (aged 30–80 years) who went through CT scans for suspected kidney masses between 2020 and 2021 [35]. Ethical approval was obtained from the KAUH IRB, and patient data were anonymized to ensure confidentiality. The dataset included 60 patients with kidney tumors (38 benign, 22 malignant) and 60 normal cases, some with cysts, stones, or hydronephrosis. Inclusion criteria were suspected kidney tumors, complete CT scan data (contrast-enhanced and non-contrast), and clinical metadata. Patients with incomplete or poor-quality imaging or unrelated diagnoses were excluded [35].

The dataset features contrast-enhanced CT scans for tumor vascularity, non-contrast scans for baseline anatomy, multiphase CT imaging (non-contrast, corticomedullary, nephrographic) for tumor differentiation, and 3D volume rendering for anatomical reconstruction. Images were converted from DICOM to JPEG format, and 70 images per patient were selected for analysis. Radiologists annotated the images, labeling them as normal, benign, or malignant, and recorded metadata such as tumor stage, location, and subtype [35].

The dataset was categorized by situation (normal, normal with cyst, tumor), tumor type (benign, malignant), subtype (adenoma, angiomyolipoma, lipoma, RCC, secondary metastasis), stage (I–IV), and location (upper, middle, lower kidney). Statistical analysis revealed that most tumors occurred in patients aged 50–70, with males being more affected than females, and that tumors were frequently located in the upper kidney region [35]. Approximately 60% of patients received contrast material while 40% did not due to contraindications. The dataset is available online using https://github.com/DaliaAlzubi/KidneyTumor (accessed on 10 April 2025).

## 4. Methods

**Figure 1:**
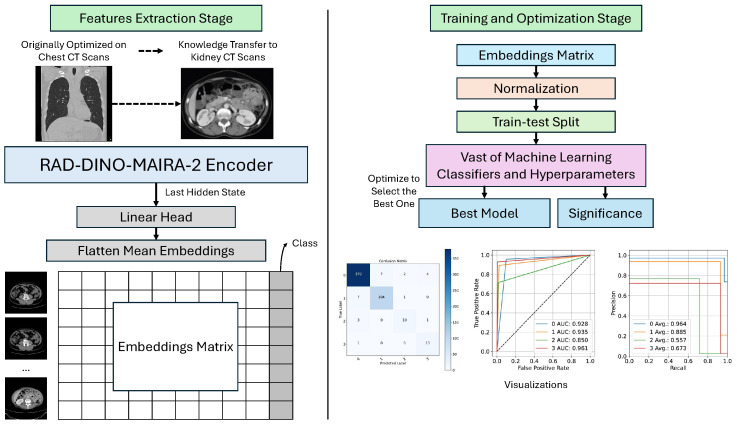
Graphical representation of the proposed kidney tumor detection and classification framework. The framework outlines the hierarchical approach, starting with feature extraction from renal CT scans, followed by data preprocessing, training, and optimization of ML classifiers. The final stage involves model assessment and visualization of results to ensure accurate and reproducible diagnostic outcomes.

The current study proposes a framework (see Figure 1) that consists of two main stages: Feature Extraction and Training and Optimization. The framework is designed to utilize advanced ML techniques for analyzing CT scans, with an anchor on kidney CT scans.

The current study begins with the Feature Extraction stage, where the process is initially optimized on chest CT scans. The knowledge gained from this optimization is then transferred to kidney CT scans, ensuring that the model benefits from prior learning. A specialized encoder, known as the RAD-DINO-MAIRA-2 Encoder, is employed to extract relevant features from the CT scans. This encoder produces two key outputs: the Last Hidden State, which represents the final hidden state from the encoder, and Flatten Mean Embeddings, which are the mean embeddings flattened for further processing. These outputs are organized into an embedding matrix for the subsequent stage.

The current study then moves to the Training and Optimization stage, where the embedding matrix is normalized to ensure consistency and improve model performance. The data is split into training and testing sets to facilitate assessment. A wide range of ML classifiers and hyperparameters are tested during optimization to identify the best-performing model. Based on this optimization, the best model is selected, and its significance is evaluated to ensure robust performance. Finally, visualizations are created to aid in interpreting and understanding the model’s results.

### 4.1. Features Extraction Stage

The process in the Features Extraction stage begins with loading the CT scan images. These images are then converted into a suitable format for processing by the RAD-DINO-MAIRA-2 encoder. The encoder, pre-trained on a large corpus of medical imaging data, processes each image to generate high-dimensional embeddings. These embeddings capture the essential features of the images, including shape, texture, and intensity patterns, which are crucial for distinguishing between different types of kidney tumors.

The RAD-DINO-MAIRA-2 encoder inherits the vision transformer (ViT) architecture, which processes the input image by dividing it into smaller patches [39]. Each one of them is linearly projected into a lower-dimensional embedding space. Let $X\in R^{H\times W\times C}$ represent the input image, where *H*, *W*, and *C* are the height, width, and number of channels, respectively. The image is split into *N* patches of size P×P, resulting in a sequence of patch embeddings $x_{p}\in R^{N\times D}$, where *D* refers to the embedding dimension. The patch embeddings are computed as in Equation (1), where $E\in R^{D\times (P^{2}\;\cdot \;C)}$ is the patch embedding projection matrix.

(1) $$x_{p}=E\;\cdot \;\mathrm{reshape}\left(X,N,P^{2}\;\cdot \;C\right)$$

To capture global information, a learnable CLS (classification) token $x_{\mathrm{cls}}\in R^{D}$ is injected into the patch embeddings sequence. The integrated sequence is then passed through a series of transformer layers with each encompassing multi-head self-attention (MSA) and feed-forward neural networks (FFN) [40]. The self-attention mechanism computes attention weights A as in Equation (2) where Q, K, and V are the query, key, and value matrices, respectively. They are obtained by linearly projecting the input embeddings. The self-attention layer output is computed as in Equation (3).

(2) $$A=\mathrm{softmax}\left(\frac{Q\;\cdot \;K^{T}}{\sqrt{D}}\right)$$

(3) MSA(x)=A·V

The final output of the RAD-DINO-MAIRA-2 encoder is the last hidden state, which includes the CLS token embedding $h_{\mathrm{cls}}$ and the patch embeddings $h_{p}$. These embeddings are used for downstream jobs (e.g., classification).

The embeddings generated by the encoder are stored in a lookup table, where each entry corresponds to a specific image. The embeddings are normalized to ensure a consistent scale, which is essential for the subsequent ML algorithms. Normalization is typically performed using techniques such as z-score normalization, which converts the data to have a zero mean (μ) and a σ=1 (standard deviation). This is presented in Equation (4) where *x* is the original embedding. Other normalization techniques, such as Min–Max scaling, may also be used depending on the dataset’s specific demands.

(4) $$z=\frac{x--\mu }{\sigma }$$

The normalized embeddings are then split into training/testing subsets, typically using an 80—20 split. This ensures that the model is evaluated on unseen data, which provides a more accurate assessment of its generalization capabilities. This stage also includes using various scalers, such as StandardScaler, MinMaxScaler, and RobustScaler, to further standardize the data before feeding it into the ML classifiers.

### 4.2. Training and Optimization

The training and optimization stage involves evaluating various ML classifiers to identify the best-performing model. The classifiers are trained on the preprocessed embeddings, and their potency is assessed using performance measures, including accuracy and F1. Optimization includes testing different hyperparameters and scalers to ensure the model performs best.

A variety of ML algorithms are utilized, including conventional classifiers such as Support Vector Machines (SVMs) and k-Nearest Neighbors (k-NN), as well as more advanced ensemble methods like Gradient Boosting and AdaBoost [41,42]. Additionally, neural network-based classifiers, such as Multi-Layer Perceptrons (MLPs), are also explored to utilize their ability to model complex, non-linear relationships in the data [43].

The aim of testing multiple ML algorithms, rather than relying on a single one, is to ensure that the chosen model is robust and well-suited to the specific characteristics of the dataset. Different algorithms have varying strengths and weaknesses; for example, SVMs are effective in high-dimensional spaces, while Random Forest handles noisy data and avoid overfitting. We can identify the algorithm that best captures the underlying patterns in the kidney CT scan data by evaluating a diverse set of classifiers.

Furthermore, this approach allows us to distinguish between the performance of conventional methods and advanced techniques, providing an understanding of whether the additional complexity of neural networks or ensemble methods is justified by improved performance. Ultimately, this comprehensive assessment ensures that the selected model is both accurate and generalizable, making it suitable for real-world medical applications.

The training process typically involves minimizing a loss function that estimates the difference between predicted and actual labels. For classification tasks, the cross-entropy loss approach is normally used. It is presented in Equation (5) where *N* is the number of samples, *C* is the classes number, $y_{i,c}$ is a binary index of whether *c* is the correct *i*-th classification, and $p_{i,c}$ is the *i*-th predicted probability belonging to *c*.

(5) $$L=-\frac{1}{N}\times \sum_{i=1}^{N}\sum_{c=1}^{C}y_{i,c}\times \log\left(p_{i,c}\right)$$

The optimization procedure focuses on adjusting hyperparameters, including learning rate, regularization intensity, and the layer count in neural networks. Grid and random search methods are frequently utilized to navigate the hyperparameter space and determine the best configuration. Furthermore, cross-validation is typically applied to verify the model’s reliability and ensure consistent performance across different data splits. The best model is determined according to its performance on the test set, and its results are visualized using ROC curves, precision–recall curves, and confusion matrices.

### 4.3. Performance Measures

The potency of the models is judged using multiple metrics, including accuracy, specificity, and F1-score. They are derived from the confusion matrix (CM), which comprehensively summarizes the model’s prediction outcomes [44,45]. The CM is a C×C matrix, where *C* represents the number of classes, and each entry $M_{i,j}$ indicates the count of samples from class *i* predicted as class *j*.

Accuracy quantifies the ratio of correctly predicted samples to the total number of samples. It is defined in Equation (6), where TP marks true positives, TN marks true negatives, FP marks false positives, and FN marks false negatives.

Precision measures the proportion of TPs among the predicted positives and is expressed in Equation (7). Recall (or sensitivity), which quantifies the proportion of TPs correctly identified, is given in Equation (8).

The F1-score reflects the precision and recall harmonic mean and provides a balanced assessment of the model’s effectiveness. It is presented in Equation (9). Specificity, which measures the ratio of TNs correctly identified, is defined in Equation (10).

(6) $$\mathrm{Accuracy}=\frac{\mathrm{TP}+\mathrm{TN}}{\mathrm{TP}+\mathrm{TN}+\mathrm{FP}+\mathrm{FN}}$$

(7) $$\mathrm{Precision}=\frac{\mathrm{TP}}{\mathrm{TP}+\mathrm{FP}}$$

(8) $$\mathrm{Recall}=\frac{\mathrm{TP}}{\mathrm{TP}+\mathrm{FN}}$$

(9) $$F1\text{-}\mathrm{score}=2\times \frac{\mathrm{Precision}\times \mathrm{Recall}}{\mathrm{Precision}+\mathrm{Recall}}$$

(10) $$\mathrm{Specificity}=\frac{\mathrm{TN}}{\mathrm{TN}+\mathrm{FP}}$$

Additionally, ROC and precision–recall curves are generated to represent the model’s performance across various thresholds visually. The ROC curve illustrates the trade-off between the true positive rate (TPR) and the false positive rate (FPR) at different threshold levels, while the precision–recall curve highlights the balance between precision and recall. The area under the ROC curve (AUC-ROC) and the area under the precision–recall curve (AUC-PRC) are robust metrics for evaluating overall model performance.

The results are saved in a history file, which records the assessment metrics for each combination of classifier and scaler. This facilitates a thorough comparison of the models and aids in identifying the most effective one. The history file typically includes key metrics such as accuracy, specificity, and F1 score for each trial, allowing for an in-depth analysis of how the models perform under different configurations.

### 4.4. The Proposed Framework Pseudocode

The proposed pseudocode framework outlines the key feature extraction, training, and optimization steps. The pseudocode is structured to reflect the workflow of the RAD-DINO-MAIRA-2 encoder, the preprocessing of embeddings, and the assessment of ML classifiers. The detailed pseudocode for the framework is shown in Algorithm 1.
**Algorithm 1:** The proposed framework pseudocode for feature extraction, training, and optimization.
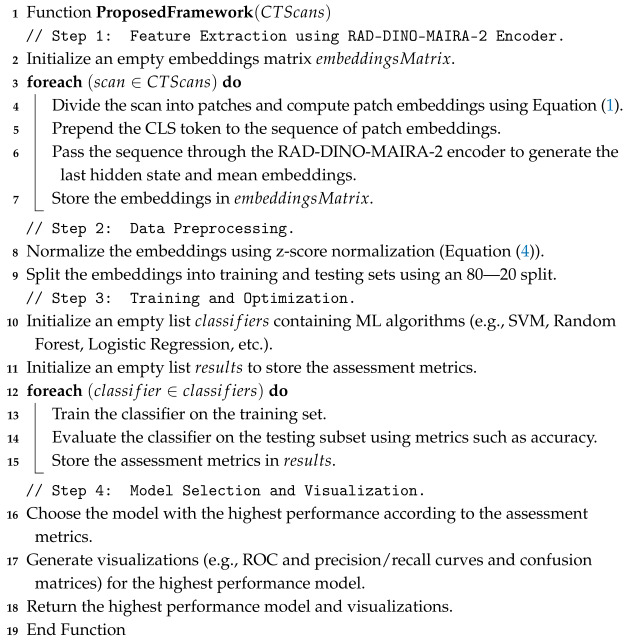


## 5. Experiments and Discussion

The current study analyzed the KAUH dataset, which comprised renal CT scans, to develop a robust kidney tumor detection and classification framework. The dataset was meticulously organized into cases and categories, structured hierarchically across four levels: normal vs. tumor, benign vs. malignant tumor, benign tumor subtypes, and malignant tumor subtypes. The primary objective was to propagate the CT scans through this hierarchical framework to arrive at a final diagnostic decision.

To achieve this, multiple experiments were conducted to identify the most effective classifier for each phase of the hierarchy, such as distinguishing between benign and malignant tumors. A total of 32 classifiers, implemented using the Scikit-Learn 1.7 Python package, were evaluated. Additionally, five optional normalization techniques were applied to the data to enhance the performance and consistency of the models.

The dataset was carefully split into training and testing sets based on case numbers rather than individual images during the learning process. This approach was adopted to prevent data leakage, ensuring that the model’s performance was evaluated on unseen cases, providing a more accurate assessment of its generalization capabilities.

After identifying the best-performing classifier for each level, the experiments were repeated 25 times to establish the confidence intervals for the results. This step was crucial for assessing the reproducibility and stability of the models. By conducting multiple trials, the study ensured that the findings were consistent and not dependent on a single run, thereby enhancing the reliability of the results. The confidence intervals provided a statistical measure of the variability in the model’s performance, offering insights into its robustness.

### 5.1. KAUH Dataset Assessment

The KAUH dataset was rigorously evaluated to assess the performance of the proposed framework for kidney tumor detection and classification. The assessment was structured across four hierarchical categories, with the analysis focusing on cases rather than individual images (each case comprised 70 images). The four categories are as follows:

- Normal vs. Tumor Classification: This category involved distinguishing between normal kidney cases and tumor cases. The dataset included 51 normal cases and 60 tumor cases.
- Tumor Type Classification: This level focused on differentiating between benign and malignant tumor cases, with 38 benign tumor cases and 22 malignant tumor cases.
- Benign Tumor Subtype Classification: This category further classified benign tumors into subtypes, including adenoma (28 cases), angiomyolipoma (8 cases), angiomyolipoma and adenomas (1 case), and lipomas (1 case).
- Malignant Tumor Subtype Classification: This level classified malignant tumors into subtypes, with 10 RCC and 12 cases of secondary subtypes.

Two sets of metrics were analyzed to evaluate the framework’s performance: the maximum performance achieved across 25 trials and the mean performance over those trials. These metrics offer beneficial information about the peak capabilities of the classifiers as well as their consistency and robustness across multiple iterations. This dual analysis ensures a comprehensive understanding of the framework’s potency in accurately classifying kidney tumors at each hierarchical level.

Table 2 presents the maximum performance metrics achieved across 25 trials for each hierarchical level. The results reveal that the framework achieved exceptional performance, with the Gaussian Process classifier and MLP classifier achieving perfect scores (100%) in tumor type classification and malignant tumor subtype classification, respectively. The Passive Aggressive classifier also performed strongly in normal vs. tumor classification, achieving an accuracy of 95.50% and an F1-score of 95.87%. The overall framework achieved a maximum accuracy of 98.29%, highlighting its peak capability in accurately classifying kidney tumors.

Table 3 presents the mean performance metrics across 25 trials for each hierarchical level. The results show that the framework consistently maintained high performance, with the Gaussian Process classifier achieving a mean accuracy of 95.82% in tumor type classification and the MLP classifier achieving a mean accuracy of 95.51% in malignant tumor subtype classification. The Passive Aggressive classifier also showed strong performance in normal vs. tumor classification, with a mean accuracy of 92.86%. The overall framework achieved a mean accuracy of 94.72%, indicating its robustness and reliability across multiple trials.

These results underscore the potency of the proposed framework in accurately classifying kidney tumors at different hierarchical levels. The high performance across both maximum and mean metrics reveals the framework’s capability to handle the complexities of kidney tumor detection and classification, making it a beneficial tool for clinical applications.

### 5.2. Rationale and Benefits of the Hierarchical Approach

The adoption of a hierarchical classification strategy in this study was driven by both clinical workflow logic and methodological advantages over flat single-stage classification models. This approach mirrors the diagnostic reasoning process employed by radiologists and clinicians, who typically first determine if a lesion is present (normal vs. tumor), then assess its nature (benign vs. malignant), and finally identify its specific subtype if malignant. By structuring the AI framework to follow this same logical progression, we enhance its interpretability and clinical alignment. The key benefits of this hierarchical design are as follows:

- Improved Model Performance and Robustness: Breaking down the complex multi-class problem into simpler sequential binary or multi-class sub-problems reduces the complexity faced by each individual classifier. For instance, distinguishing between normal tissue and any type of tumor (Level 1) is a more straightforward task than directly classifying among all possible subtypes simultaneously. This simplification often leads to higher accuracy at each level, as demonstrated by our results where classifiers achieved perfect scores (100%) in specific sub-tasks like tumor type and malignant subtype classification. The overall framework’s high mean accuracy (94.72%) and maximum accuracy (98.29%) across 25 trials attest to the robustness gained from this staged approach.
- Enhanced Clinical Utility and Decision Support: A hierarchical output provides clinicians with a structured diagnostic pathway. Instead of a single, potentially opaque prediction, the system offers intermediate results (e.g., “Tumor Detected”, then “Malignant”, then “RCC”). This step-by-step output is more intuitive for medical professionals, allowing them to understand the model’s reasoning and build trust. It also facilitates targeted follow-up; for example, if the model identifies a benign tumor, further invasive procedures might be avoided, while a malignant prediction triggers immediate referral for specialized oncology care.
- Increased Interpretability and Trust: Each level of the hierarchy can be analyzed independently. This allows for easier identification of potential failure modes (e.g., if Level 1 performs well but Level 2 struggles, it suggests an issue with benign/malignant differentiation). Furthermore, the use of different best-performing classifiers at each level (Passive Aggressive for Level 1, Gaussian Process for Level 2, etc.) allows us to leverage the strengths of various algorithms for specific tasks, potentially increasing overall reliability. This modularity makes the system more transparent than a monolithic deep learning model.
- Mitigation of Class Imbalance Effects: In datasets like KAUH, some classes (e.g., specific benign subtypes) may have very few samples. A flat multi-class model can be heavily biased towards majority classes. The hierarchical approach mitigates this by isolating smaller classes into later stages where they are compared against fewer alternatives. For example, distinguishing between adenoma and angiomyolipoma (Level 3) involves only those two classes, rather than competing against all other categories in a single model.
- Scalability and Flexibility: The hierarchical structure is inherently scalable. New tumor subtypes can be added to existing levels without retraining the entire model. Similarly, if future research identifies a new biomarker relevant to a specific stage, the corresponding classifier can be updated or replaced independently, making the framework adaptable to evolving medical knowledge.

With that said, the hierarchical ML framework is not merely a technical choice but a strategic one designed to align with clinical practice, improve model performance through task decomposition, enhance interpretability for end-users, and provide a flexible platform for future development. This structure directly addresses the gap identified in the Introduction regarding the lack of granularity in current classification systems and the need for clinically viable, interpretable tools.

### 5.3. Statistical Analysis

**Figure 2:**
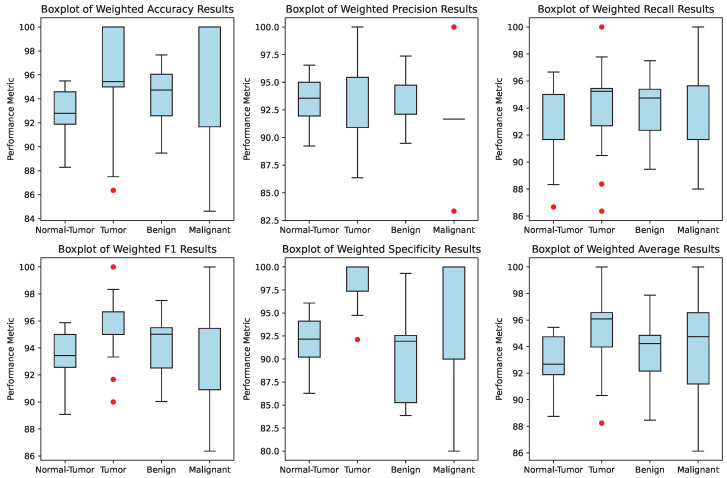
Box plot of performance metrics across 25 trials for each hierarchical level. The plot illustrates the distribution of accuracy, specificity, and F1, demonstrating the consistency and variability of the model’s performance (Red dots depict the performance outliers).

Statistical analyses were conducted to validate the proposed framework’s robustness further. Figure 2 presents a box plot of all performance metrics across the 25 trials for each hierarchical level. The box plot illustrates the distribution of accuracy, specificity, and F1, highlighting the consistency and variability of the model’s performance. The narrow interquartile ranges and minimal outliers indicate that the framework is highly stable and reproducible.

**Figure 3:**
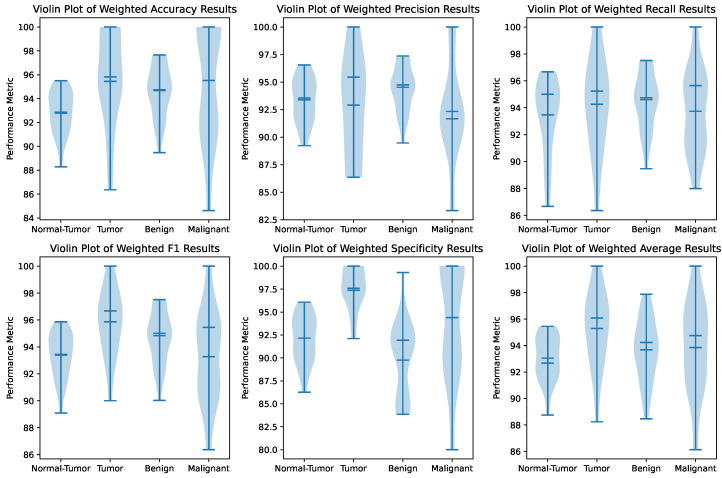
Violin plot of performance metrics across 25 trials for each hierarchical level. The plot provides a detailed view of the data distribution, confirming the high density of performance metrics around the median values and the framework’s reliability.

Additionally, Figure 3 provides a violin plot of the same metrics, offering a more detailed view of the data distribution. The violin plot confirms the high density of performance metrics around the median values, further supporting the framework’s reliability. The symmetrical shapes of the violin plots indicate that the performance metrics are evenly distributed, with no significant skewness.

These statistical analyses underscore the robustness and reproducibility of the proposed framework, making it a promising tool for kidney tumor detection and classification in clinical settings. The model’s high accuracy, consistency, and stability across multiple trials suggest that it can be effectively integrated into medical imaging workflows to assist radiologists in making accurate and timely diagnoses.

### 5.4. Related Studies Comparisons

The current study builds upon the methodologies and findings of the aforementioned studies while introducing several novel contributions. Table 4 is a comparison table summarizing the key aspects of each study.

The current study achieved a mean accuracy of 94.72% and a maximum accuracy of 98.29% across 25 independent trials. These results reveal the model’s high reproducibility and robustness, with narrow confidence intervals as demonstrated by the box plots in Figure 2 and violin plots in Figure 3. This rigorous assessment process provides a statistically sound measure of performance variability, ensuring that the findings are consistent and not dependent on a single run.

In contrast, previous studies typically report single-run accuracies or validation set performance without providing confidence intervals or measures of variance. For instance, Alzu et al. [35] report a 92% classification accuracy, but this value represents a single evaluation on their test set. Similarly, Praveen et al. [34] report a 94% accuracy on a subset of the KAUH dataset, and Kaur et al. [38] report training and validation accuracies, but none of these studies quantify the uncertainty or stability of their results through repeated trials.

Our approach of conducting 25 trials offers a more comprehensive and reliable evaluation. The mean accuracy of 94.72% reflects the framework’s stable performance, while the maximum accuracy of 98.29% demonstrates its peak capability. The low variance observed in Figure 2 and Figure 3 confirms that the improvements over prior work are consistent and statistically significant, rather than being artifacts of random initialization or data splitting. This level of statistical analysis is crucial for establishing the reliability of an AI model intended for clinical use, where consistent performance is paramount.

### 5.5. Addressing Interpretability Through Hierarchical Design and Future Directions

A significant limitation of many DL models in medical imaging is their “black-box” nature, which hinders clinician trust and adoption. Our framework directly addresses this gap through its hierarchical design and the strategic selection of interpretable classifiers at each level.

Inherent Interpretability via Hierarchical Reasoning: The primary source of interpretability lies in the framework’s architecture. Instead of producing a single, opaque prediction, it generates a sequential, multi-step output (e.g., “Normal vs. Tumor → Benign vs. Malignant → RCC”). This mimics the logical diagnostic pathway used by radiologists, making the AI’s decision process transparent and easy to follow. Clinicians can understand why a final diagnosis was reached by reviewing the intermediate decisions, building confidence in the system’s reliability.

Utilizing Classifier-Specific Explainability: At each hierarchical level, we selected the following classifiers known for their interpretability or potential for explanation.

- Gaussian Process (Level 2–Tumor Type): GP models inherently provide uncertainty estimates (variance) alongside predictions. High uncertainty scores for a specific case can flag it for human review, acting as a built-in “confidence indicator” for clinicians.
- MLP (Level 4–Malignant Subtype): While traditionally considered less interpretable, modern techniques can be applied to MLPs. We plan to utilize methods like Layer-wise Relevance Propagation (LRP) or Integrated Gradients to identify which input features (i.e., which parts of the CT scan embeddings) contributed most to the final classification decision.
- Passive Aggressive (Level 1–Normal vs. Tumor): As a linear model variant, Passive Aggressive classifiers can provide weights for each input feature. Analyzing these weights can reveal which embedding dimensions (derived from the RAD-DINO-MAIRA-2 encoder) are most discriminative for detecting the presence of any tumor.

Future Work for Enhanced Interpretability: To further strengthen the interpretability of our framework, we propose the following future developments.

- Integration of Visualization Techniques: We will implement Grad-CAM (Gradient-weighted Class Activation Mapping) or similar attention mechanisms on the RAD-DINO-MAIRA-2 encoder’s outputs. This would generate heatmaps overlaid on the original CT images, highlighting the specific anatomical regions that influenced the classifier’s decision at each hierarchical level. This provides direct, visual evidence for the AI’s reasoning.
- SHAP (SHapley Additive exPlanations) Analysis: Applying SHAP values to the final prediction (or intermediate predictions) will quantify the contribution of each input feature (embedding dimension) to the output. This offers a global understanding of which features are most important across the entire dataset and can also explain individual predictions.
- Clinical Feedback Loop: We envision a future version of the system where clinicians can provide feedback on the AI’s predictions and highlighted regions. This feedback can be used to refine the model’s focus areas and improve the alignment between AI-generated explanations and clinical intuition.

With that said, our framework moves beyond the black-box paradigm. It aims to provide clinicians with not just a diagnosis, but also understandable reasons for that diagnosis, thereby fostering trust and facilitating its integration into real-world clinical workflows.

## 6. Conclusions and Future Work

This study presents a robust and hierarchical AI-driven framework for detecting and classifying kidney tumors using CT scans. By utilizing advanced ML techniques and a specialized encoder (RAD-DINO-MAIRA-2), the framework achieves high accuracy and consistency in classifying kidney tumors at multiple hierarchical levels. The results reveal the potential of AI to address the limitations of conventional diagnostic methods, such as intra-observer and inter-observer variability, and improve kidney tumor diagnosis’s efficiency and reliability. The framework’s performance is highly reproducible, with a maximum accuracy of 98.29% and a mean accuracy of 94.72% across 25 trials. The Gaussian Process classifier and MLP classifier achieved perfect scores in tumor type classification and malignant tumor subtype classification, respectively. These results underscore the potency of the hierarchical approach in handling the complexities of kidney tumor detection and classification, making it a beneficial tool for clinical applications. Overall, this study highlights the transformative potential of AI in medical imaging, particularly in the context of kidney tumor diagnosis. By providing a reliable and accurate diagnostic tool, the framework has the potential to enhance early detection, improve patient outcomes, and reduce the diagnostic burden on radiologists. This study also highlights the usefulness of reproducibility and robustness in AI models, ensuring that they can be effectively integrated into clinical workflows.

Future works may concentrate on extending the dataset to incorporate more diverse populations and imaging methods, refining the framework for real-time clinical applications, and improving model interpretability to guarantee transparency and reliability in clinical environments. Crucially, to validate the framework’s external generalizability, we will undertake multi-center studies using independent datasets from collaborating institutions. This will involve rigorous external validation and, if necessary, the application of domain adaptation techniques to ensure robust performance across diverse clinical settings. Furthermore, external validation using independent datasets and the consideration of ethical issues of data privacy and algorithmic bias will be essential for the proper implementation of AI in healthcare.

## Figures and Tables

**Table 1:** Mapping between identified research gaps and the corresponding contributions of the proposed framework.

Identified Research Gap	Corresponding Contribution/Resolution in This Work
1. Dataset variability and bias—Most existing models are trained on limited or homogeneous datasets, reducing generalizability to real clinical cases.	Robust, multi-trial validation (25 independent runs) across benchmark kidney tumor datasets ensures consistent results and improved generalization to diverse patient data.
2. Model robustness and reproducibility—Reported accuracies fluctuate across trials, showing instability and low reproducibility.	Hierarchical multi-classifier evaluation with statistical validation of performance (accuracy, robustness, reproducibility metrics) provides stable and reproducible diagnostic outcomes.
3. Clinical integration barriers—Many frameworks lack workflow-compatible design, hindering translation into hospital environments.	Clinically aligned design that integrates hierarchical diagnosis steps resembling the actual clinical workflow, making the system suitable for deployment in radiological settings.
4. Granularity of classification—Existing models often stop at binary (benign vs. malignant) classification, ignoring malignant subtype differentiation.	Two-tier hierarchical classification enabling both tumor type and malignant subtype prediction, directly addressing clinical needs for personalized treatment planning.
5. Interpretability and trust—Lack of explainable and transparent decision mechanisms limits clinician confidence.	Interpretable hierarchical structure and explainable models (e.g., Gaussian Process, MLP) facilitate clearer visualization and trust in diagnostic reasoning.
6. Limited feature extraction adaptability—Previous encoders fail to capture the fine-grained texture details of kidney CT scans.	Development of RAD-DINO-MAIRA-2 encoder, specialized for discriminative medical imaging features, enhancing feature quality and downstream classification performance.

**Table 2:** Performance metrics of the best-performing classifiers for each hierarchical level in the KAUH dataset. The table presents weighted metrics and average performance metrics (max of the 25 trials) for normal vs. tumor classification, tumor type classification, benign tumor subtype classification, and malignant tumor subtype classification.

Category	Classifier	Scaler	Accuracy	Precision	Recall	F1	Specificity	Average
Normal vs. Tumor	Passive Aggressive	Robust	95.50%	96.55%	96.67%	95.87%	96.08%	95.45%
Tumor Types	Gaussian Process	Quantile	100.00%	100.00%	100.00%	100.00%	100.00%	100.00%
Benign Tumor Types	Kneighbors	Quantile	97.66%	97.37%	97.51%	97.51%	99.30%	97.87%
Malignant Tumor Types	MLP	Robust	100.00%	100.00%	100.00%	100.00%	100.00%	100.00%
Overall Framework			98.29%	98.48%	98.55%	98.34%	98.84%	98.33%

**Table 3:** Performance metrics of the best-performing classifiers for each hierarchical level in the KAUH dataset. The table presents weighted metrics and average performance metrics (mean of the 25 trials) for normal vs. tumor classification, tumor type classification, benign tumor subtype classification, and malignant tumor subtype classification.

Category	Classifier	Scaler	Accuracy	Precision	Recall	F1	Specificity	Average
Normal vs. Tumor	Passive Aggressive	Robust	92.86%	93.39%	93.47%	93.39%	92.16%	93.05%
Tumor Types	Gaussian Process	Quantile	95.82%	92.91%	94.26%	95.87%	97.58%	95.29%
Benign Tumor Types	Kneighbors	Quantile	94.67%	94.53%	94.60%	94.83%	89.77%	93.68%
Malignant Tumor Types	MLP	Robust	95.51%	92.33%	93.74%	93.27%	94.40%	93.85%
Overall Framework			94.72%	93.29%	94.02%	94.34%	93.48%	93.97%

**Table 4:** Comparison of related studies and the current study: A summary of methodologies, datasets, model architectures, accuracy metrics, and key contributions across studies focusing on kidney tumor detection and classification using the KAUH dataset and similar datasets.

Study	Year	Dataset	Model Architecture	Accuracy	Key Contributions
Alzu et al. [35]	2022	8400 images (KAUH)	CNN-6, ResNet50, VGG16, CNN-4	97%, 96%, 60%, 92%	Development of multiple 2D-CNN models for detection and classification.
Praveen et al. [34]	2023	2170 images (a subset of KAUH)	ResNet + ResNeXt	94%	Fusion of ResNet and ResNeXt for improved gradient flow and scalability.
Kaur et al. [38]	2024	8400 images	Sequential CNN	97.69% (train), 95.31% (val)	Use of data augmentation and a well-structured CNN for high precision and recall.
Current Study	2025	8400 images (KAUH)	Hierarchical Framework (32 classifiers)	94.72% (mean), 98.29% (max)	Hierarchical classification, 25 trials for reproducibility, and use of 32 classifiers.

## Data Availability

The dataset is available at: https://github.com/DaliaAlzubi/KidneyTumor (accessed on 10 April 2025).
